# Cross-plane transport in a single-molecule two-dimensional van der Waals heterojunction

**DOI:** 10.1126/sciadv.aba6714

**Published:** 2020-05-29

**Authors:** Shiqiang Zhao, Qingqing Wu, Jiuchan Pi, Junyang Liu, Jueting Zheng, Songjun Hou, Junying Wei, Ruihao Li, Hatef Sadeghi, Yang Yang, Jia Shi, Zhaobin Chen, Zongyuan Xiao, Colin Lambert, Wenjing Hong

**Affiliations:** 1 State Key Laboratory of Physical Chemistry of Solid Surfaces, iChEM, College of Chemistry and Chemical Engineering & Pen-Tung Sah Institute of Micro-Nano Science and Technology, Xiamen University, Xiamen 361005, China.; 2 Department of Physics, Lancaster University, Lancaster LA1 4YB, UK.; 3 Innovation Laboratory for Sciences and Technologies of Energy Materials of Fujian Province (IKKEM), Xiamen University, Xiamen 361005, China.

## Abstract

Two-dimensional van der Waals heterojunctions (2D-vdWHs) stacked from atomically thick 2D materials are predicted to be a diverse class of electronic materials with unique electronic properties. These properties can be further tuned by sandwiching monolayers of planar organic molecules between 2D materials to form molecular 2D-vdWHs (M-2D-vdWHs), in which electricity flows in a cross-plane way from one 2D layer to the other via a single molecular layer. Using a newly developed cross-plane break junction technique, combined with density functional theory calculations, we show that M-2D-vdWHs can be created and that cross-plane charge transport can be tuned by incorporating guest molecules. The M-2D-vdWHs exhibit distinct cross-plane charge transport signatures, which differ from those of molecules undergoing in-plane charge transport.

## Teaser

The cross-plane charge transport is demonstrated in single-molecule two-dimensional van der Waals heterojunctions.

## INTRODUCTION

**Fig. 1:**
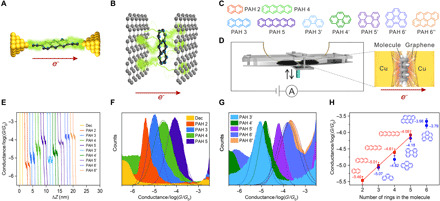
Fabrication and charge transport characterization of graphene M-2D-vdWHs. Illustrations of in-plane (**A**) and cross-plane (**B**) charge transport. Green trajectories are indicative of electron scattering paths. (**C**) Chemical structures of the polycyclic aromatic hydrocarbons (PAHs) that sandwiched between two graphene electrodes. In particular, naphthalene (PAH 2), anthracene (PAH 3), tetracene (PAH 4), and pentacene (PAH 5) are linear PAHs, while phenanthrene (PAH 3′), pyrene (PAH 4′), perylene (PAH 5′), benzoperylene (PAH 6′), and anthanthrene (PAH 6″) are nonlinear PAHs. (**D**) Schematic of the cross-plane break junction (XPBJ) technique and the device structure of the studied graphene M-2D-vdWHs. (**E**) Examples of conductance versus displacement traces measured with PAHs and traces measured without the PAHs (yellow, Dec). The traces are shifted horizontally for clarity. 1D conductance histograms generated from ~1000 traces for graphene M-2D-vdWH of the linear PAHs (**F**) and the nonlinear PAHs (**G**). (**H**) The single-molecule conductance of each graphene M-2D-vdWH, plotted as a function of the number of benzene rings of the sandwiched molecule. The error bar is determined from the chip-to-chip variation of three independent experiments.

The wide variety of currently available two-dimensional (2D) materials has enabled the stacking of different atomic layers to yield new electronic materials held together by van der Waals (vdW) forces (*1*–*5*). Despite their early promise (*6*), the preparation of defect-free 2D-vdW heterojunctions (2D-vdWHs) remains a challenge. To date, 2D-vdWHs have been fabricated using top-down (exfoliation and restacking) and bottom-up [chemical vapor deposition (CVD) growth] approaches (*7*, *8*). However, the mechanical transfer of 2D materials is time-consuming and remains technically challenging, while the CVD growth of one 2D material on another requires sophisticated techniques and strict growth conditions (*9*). Methodologies developed for single-molecule electronics offers a unique opportunity for creating molecular 2D-vdW heterojunctions (M-2D-vdWHs), in which selected molecules are sandwiched between the two 2D material layers and stabilized by vdW interactions (*10*). For several years, break junction techniques (*11*–*14*) have been used to trap single molecules between two gold electrodes with subangstrom precision. In previous experiments, the molecule is bonded to the electrodes by terminal anchor groups at two ends of the molecule and electron transport through the molecular junction with an in-plane way (Fig. 1A). In M-2D-vdWHs developed in this work, electrons can transport through the junction in a cross-plane way, i.e., perpendicular to the planes of the 2D materials based on vdW interaction (Fig. 1B) (*15*). Because of the large molecular library (*16*–*18*) and the variety of responses of molecules to external stimuli (*19*–*22*), such a transport mode can open new routes for the fine tuning of charge transport through M-2D-vdWHs. Nevertheless, to date, there are no experimental measurements of the cross-plane charge transport due to the lack of a method for fabricating precise M-2D-vdWHs.

## RESULTS

### Fabrication and charge transport measurement of graphene-molecule-graphene 2D-vdWHs

To achieve this goal, we develop a cross-plane break junction (XPBJ) technique to fabricate well-defined M-2D-vdWHs with atomic thickness. The XPBJ method was used to construct a large number of graphene-molecule-graphene 2D-vdWHs (graphene M-2D-vdWHs) by repeatedly opening and closing a graphene electrode pair in solution. During the mechanical manipulation of the graphene electrode pair, the cross-plane current was recorded by a current-voltage (*I*-*V*) converter for further statistical analysis. A family of polycyclic aromatic hydrocarbons (PAHs) was selected as the probe molecules (Fig. 1C). Benefiting from their planar structures and the conjugated component therein, these PAHs can couple with graphene electrode and thus allow the fabrication of M-2D-vdWHs (Fig. 1D). These PAHs are of general interest in molecular electronics because the molecular orbital energy level of them can be tuned by changing the number of phenyl rings (*23*, *24*). This XPBJ technique allows the unambiguous and reproducible characterization of charge transport through the cross-plane interfaces of the graphene M-2D-vdWHs and demonstrates that cross-plane charge transport through M-2D-vdWHs can be tuned by incorporating diverse molecules.

To investigate charge transport through the M-2D-vdWHs, both the microchip and the operational mechanics of the mechanically controllable break junction (MCBJ) (*25*) were redesigned to realize the XPBJ. Two Cu wires coated with CVD-grown single-layer graphene were bent to be O-ring shape, placed in close proximity (~10-μm separation), and fixed on an elastic substrate to serve as the microchip of the break junction technique. During XPBJ measurement, the two graphene electrodes were immersed in a solution containing the target molecules and brought into contact with each other by a downward bending of the substrate. The direction of the actuator movement was switched when the measured conductance reached either a high conductance value of 10^−2.5^ *G*_0_ (where *G*_0_ is quantum conductance, ~245.4 nS) or a low conductance value of 10^−7.5^ *G*_0_ (~2.45 pS). The pre-set high value prevents damage of the graphene layer or the formation of a Cu-Cu contact, while the low value prevents the recording of insignificant noise data. Figure 1E shows the individual conductance traces recorded with this modified MCBJ technique (fig. S2) using a 100-mV bias voltage in solutions containing the target molecules and that recorded in the pure solvent. For the pure solvent, the individual conductance traces contain no discernible plateau (Fig. 1E). In contrast, in the presence of PAH molecules, there emerged clear plateaus in the conductance traces in the conductance range between 10^−3^ *G*_0_ (~77.6 nS) and 10^−6^ *G*_0_ (~0.1 nS). To obtain the statistics, we collected more than 1000 conductance traces for pure solvent and each of the PAH molecules. The plateaus contributed to the distinct peaks in their corresponding 1D conductance histograms (Fig. 1, F and G) and the intensified conductance clouds in 2D conductance-distance histograms (fig. S4), suggesting the formation of single graphene-molecule-graphene junctions (*12*). It is found that PAH 6″ provides the highest conductance at 10^−3.66^ *G*_0_ (~17.0 nS), which is about ~60 times higher than the lowest conductance from PAH 2 at 10^−5.46^ *G*_0_ (~0.3 nS).

To investigate the dependence on the cross-plane area of these molecular junctions, we plotted the measured conductances as a function of the number of benzene rings, as shown in Fig. 1H. In conventional in-plane single-molecule junctions, where the current flows in the plane of the molecular backbone, the conduction decreases when the length of the molecule increases because the molecules act as tunnel barriers (*26*, *27*). In contrast, in our experiments, the shortest molecule PAH 2 has the lowest conductance (Fig. 1H). This observation shows that the geometries of the as-fabricated graphene-molecule-graphene junctions are M-2D-vdWHs, where the target molecules lie flat on the surface of the graphene electrode and create a cross-plane conduction path (Fig. 1D, red arrows). With this framework, an increase in conductance with the number of benzene rings is expected (Fig. 1, F and G), because of the corresponding increase in the cross-plane area. Classically, if Ohm’s law is applicable, the conductance should scale with the number of benzenes. However, the measured exponential dependence of the conductance on the number of benzene rings for linear PAH molecules is completely nonclassical, and, notably, for two PAH molecules with the same number of benzene rings, their conductances are different (Fig. 1H). For instance, the conductance of PAH 4 (10^−4.61^ *G*_0_, ~1.9 nS) is around 50% higher than that of PAH 4′ (10^−4.82^ *G*_0_, ~1.2 nS), molecular topology–dependent conductance further suggests room-temperature quantum transport.

### Determine the microscopic structures of the graphene M-2D-vdWHs

**Fig. 2:**
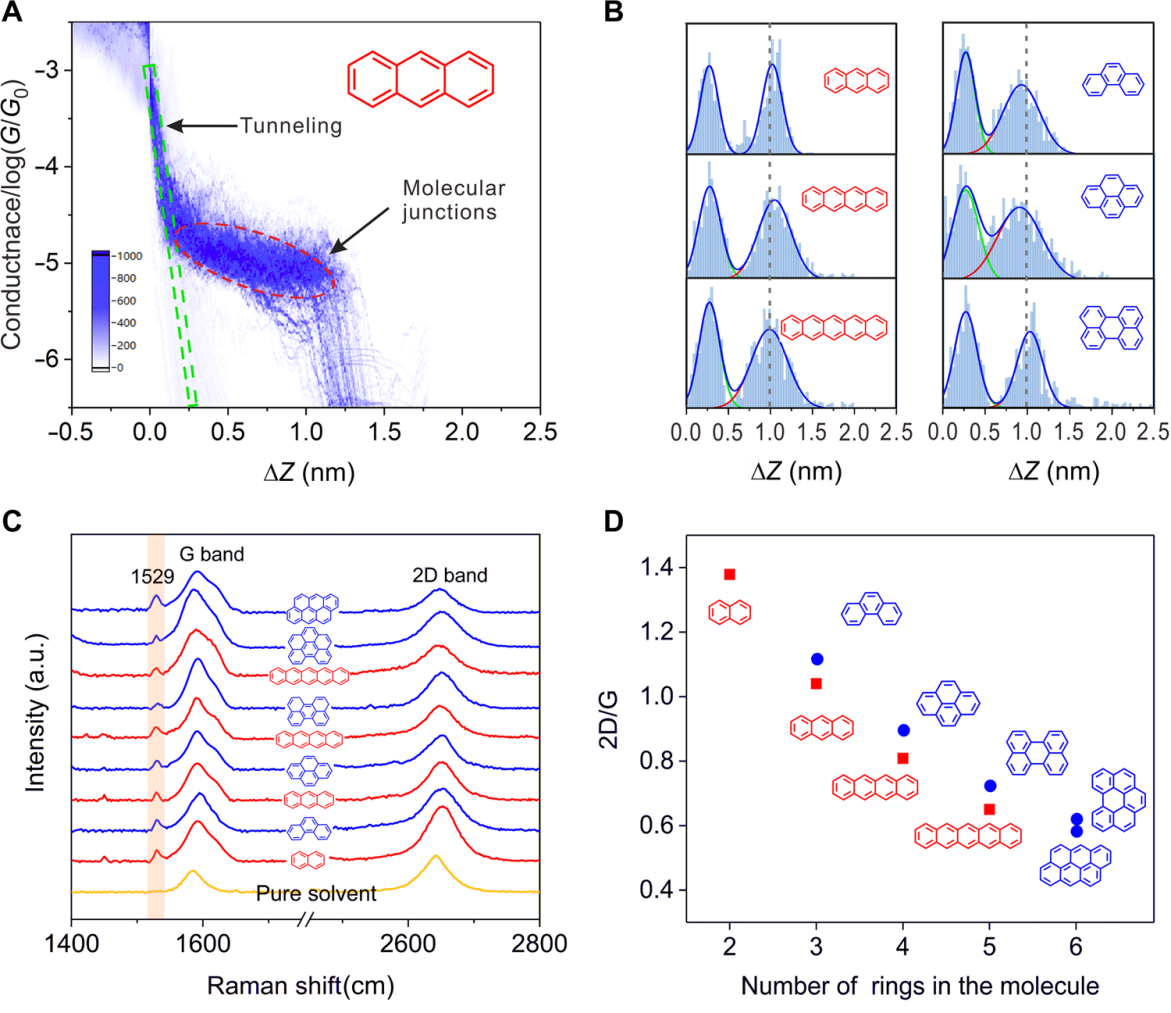
Displacement and Raman characterization of the graphene M-2D-vdWHs. (**A**) The 2D conductance-distance histogram for the graphene M-2D-vdWHs with PAH 3. The green rectangular dashed frame represents tunneling in decane, and the red elliptical dashed frame represents molecular junctions. (**B**) The relative displacement distributions for the graphene M-2D-vdWHs with PAH 3, PAH 4, PAH 5, PAH 3′, PAH 4′, and PAH 5′. (**C**) Raman spectrum of the graphene electrode pair that experienced XPBJ operation in the presence of pure solvent (the yellow curve), and the Raman spectra of the graphene M-2D-vdWHs fabricated by the XPBJ method. The sandwiched molecules of the graphene M-2D-vdWHs are PAHs. a.u., arbitrary units. (**D**) The ratio of the intensities of the G and 2D peaks as a function of the number of benzene rings.

To determine the microscopic structures of the M-2D-vdWHs, we constructed the 2D conductance-distance histogram for the graphene M-2D-vdWHs with PAHs and then statistically analyzed their single-molecule conductance features (*28*). Figure 2A gives the 2D histogram for the graphene M-2D-vdWHs with PAH 3, while Fig. 2B shows the displacement distributions for six typical graphene M-2D-vdWHs. The measurement results for the pure solvent and all the other PAHs are given in figs. S5 and S7. In comparison with the control experiment of pure solvent, it was inferred that the peak at about 0.27 nm arises from the direct tunneling between the two graphene electrodes (*29*), and the latter one is attributed to the graphene M-2D-vdWHs with PAH 3. As PAH 3 can be viewed as a graphene nanoribbon, the thickness of PAH 3 is approximately the same as the single-layer graphene sheet (~1.0 nm) (*30*), which agrees well with the probable plateau length measured in our experiment. The most probable plateau lengths for the PAH molecules lie in 0.87 to 1.08 nm (Fig. 2B and fig. S6, A to C). These values are considerably smaller than the molecular lengths of the PAH molecules and comparable with the spacing between two graphene layers incorporated with PAH molecules (fig. S6 and table S2). This observation verified the cross-plane model of charge transport in the graphene M-2D-vdWHs (Fig. 1B). For each probe molecule, we performed the measurement in at least three microchips. Figures S8 and S9 give two other sets of the constructed histograms, which show consistent results. Moreover, we performed the single-molecule conductance measurement with different concentrations. As shown in fig. S10, the formation probability of M-2D-vdWHs is dependent on the concentration, while the measured conductance value is independent of the concentration, which shows that the conductance characterization comes from single probe molecule.

To further understand the microscopic configurations of the as-fabricated junctions, Raman spectroscopy (*31*) was used to investigate the molecule assembled on graphene electrodes. For each of the other spectra acquired in the presence of the target molecules, there is a significant peak at 1529 cm^−1^ (Fig. 2C). Neither the graphene nor the target molecule alone exhibits this peak (fig. S11). Thus, this signal is attributed to the adsorption of target molecules on the graphene surface. A previous report demonstrated that this signal is also an indicator of single-layer graphene because this signal is absent in the Raman spectra in the case of aromatic molecules absorbed on graphite or multilayer graphene (*32*). The ratio of the intensities of the G and 2D peaks as a function of the number of benzene rings was plotted in Fig. 2D. This ratio is determined by the electron concentration of graphene after doping and therefore shows the level of doping (*33*); thus, the decreasing curve indicates that the degree of charge transport between each target molecule and the graphene electrode increases as the number of benzene rings increases (Fig. 2D), which agrees well with the path analysis of the graphene M-2D-vdWHs depicted in Fig. 1H. To assess whether the graphene layer is able to retain itself during the XPBJ experiments, we had characterized the graphene electrodes before and after XPBJ experiments (fig. S11). It is found that in the two Raman spectra, the 2D peaks are quite sharp and there is no discernible difference between them (see table S3 for further information), which indicates that there is a high-quality graphene layer on the copper wires, all through the XPBJ operation (*34*).

### Density functional theory simulations

**Fig. 3:**
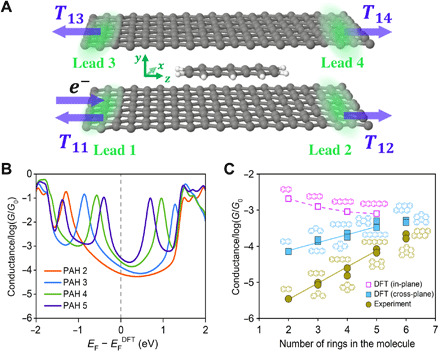
Theoretical simulations for the charge transport in the graphene M-2D-vdWHs. (**A**) Schematic of the graphene M-2D-vdWHs, where PAH 3 is sandwiched between two graphene sheets, used in the calculations. Electrons are injected from lead 1. *T*_11_ is the reflection coefficient, while *T*_12_, *T*_13_, and *T*_14_ are transmission coefficients into the other three terminals. (**B**) Boltzmann-weighted average conductances collected from the gray curves of fig. S14 as a function of the Fermi level relative to that predicted by density functional theory (DFT). The Fermi energy is depicted by the gray vertical dashed line, which is estimated by DFT. Those average conductances are calculated on the basis of binding energies and derived from transmission functions (obtained from the sum of the transmission coefficients *T*_13_ + *T*_14_) by eqs. S1 to S6 of section S6. The results of linear PAHs are displayed for a tidy and clear view, while those of all the PAHs are shown in fig. S14. (**C**) Conductances at Fermi energy from DFT calculations and those from the experiment. The brown dots show the experimental values, while the light blue dots represent the theoretical results for graphene M-2D-vdWHs junctions. The magenta is the conductance of linear PAHs in gold-gold break junctions with in-plane transport (fig. S16).

To elucidate the nonlinear conductances increase in graphene M-2D-vdWHs with the number of benzene rings, we calculated their cross-plane conductances using a combination of the ab initio density function theory package SIESTA (*35*) and the quantum transport code Gollum (*36*). Figure 3A shows the model of cross-plane graphene M-2D-vdWHs used in the calculations. Each sheet extends to ±infinity in the *z* direction, and the structure is regarded as a four-probe device, in which the cross-plane current injected from lead 1 is collected by leads 3 and 4. To avoid edge effects, the graphene sheets are assigned periodic boundary conditions in the *x* direction. The PAHs lie between the two graphene sheets with a separation varying from 3.3 to 3.5 Å depending on the AA, AB, or intermediate nature of the stacking between the sandwiched molecule and the graphene sheets (figs. S12 and S13). We calculated the binding energy *E*_b_ for these stacking configurations using a basis set superposition error correction (*37*, *38*), where *E*_b_ = *E*[AB] − *E*[Ab] − *E*[aB], and found that AB stacking is the most favorable configuration (see fig. S14 for further details). The distance between the adsorbed molecule and graphene and the magnitude of binding energy are in qualitative agreement with the literature values (*39*).

After extracting the resulting mean-field Hamiltonian and overlap matrices, we computed the electrical properties of the devices using the quantum transport code Gollum (more details can be seen in Materials and Methods) (*36*). Figure 3B shows the calculated average conductances of linear PAHs stemming from the electron transport from lead 1 to leads 3 and 4 as a function of the Fermi energy *E*_F_. More details and conductances of other PAHs are shown in figs. S14 and S15. The conductances are weighted by Boltzmann factors based on binding energies of AA stacking, AB stacking, and one intermediate stacking configuration (see figs. S12 and S13 and section S6). In agreement with our experimental values, the conductances for linear PAHs (PAH 2 to PAH 5) increase approximately exponentially with the number of benzene rings over a wide range of Fermi energies *E*_F_ near the density functional theory (DFT)–predicted Fermi energy *E*_F_^DFT^ of a pristine graphene sheet. Although the precise value of *E*_F_ depends on the doping of the graphene in contact with the Cu wires, qualitative agreement with the experimental result was obtained even at the ideal DFT-predicted value. The agreement between the calculated (light blue dots) and the measured (brown dots) conductance values shows clear evidence that cross-plane transport is quantum mechanical in nature and takes place via phase-coherent tunneling. Furthermore, we find that different shapes of the same area lead to different conductances (Fig. 3C). These findings are in contrast with a classical picture of cross-plane transport, where the conductance is expected to increase in proportion to the area or, equivalently, the number of benzene rings. The geometries of M-2D-vdWHs are entirely different from junctions fabricated with conventional metallic electrodes. In the case of PAHs, it was recently observed that the molecular junctions adopt “sandwich compound” compact geometries when using either Ag or Pt electrodes (*24*). However, although the sandwiched molecules there are also flat, the PAHs in these devices bind to the metallic electrodes with a covalent bond and exhibit conventional in-plane transport. It is also reported that the conductance decreases from naphthalene to anthracene with thiophenyl anchor in traditional MCBJ that involved with Au electrodes (*40*). Similar results are reproduced in our in-pane calculations shown by the magenta dots in Fig. 3C (see fig. S16 for details). In contrast, in the M-2D-vdWHs studied here, the PAHs couple electronically to the graphene electrodes via π-π stacking interactions and electricity flows via cross-plane transport, in which all the components of the sandwiched molecules are active, i.e., all the benzene rings are directly coupled to the graphene electrodes.

## DISCUSSION

To conclude, we report the fabrication of single 2D-vdW heterojunctions with atomic thickness using a newly developed XPBJ technique. Thousands of graphene M-2D-vdWHs were repeatedly fabricated, allowing the measurement of cross-plane charge transport through vdW heterojunction with 2D materials. Using a family of PAHs as model molecules, we found that their cross-plane charge transport is distinct from the conventional in-plane charge transport. In contrast with conventional single-molecule junctions with terminal groups for binding the molecule to electrodes, no terminal groups are needed for measurements of cross-plane transport. This is advantageous because the insertion of terminal groups involves additional synthesis and limits the range of molecules that can be measured in conventional junctions and can significantly affect the locations of frontier orbitals relative to the Fermi energy of electrodes. Comparison between theory and experiment demonstrates that room-temperature cross-plane transport is quantum mechanical, takes place via phase-coherent tunneling, and involves π-π overlap between the electrodes and all the benzene rings in the molecules. This result suggests that in the future, strategies based on tuning the electronic properties of sandwiched molecules can be used to control cross-plane transport. The 2D-vdWHs devices and the versatile fabrication technique developed in this work can be extended to various molecular materials and opens new opportunities for exploiting the chemistry, design, fabrication, and characterization of molecular 2D materials with vdW heterojunctions.

## MATERIALS AND METHODS

### Materials

The Cu wires that coated with CVD-grown single-layer graphene were purchased (6 Carbon Technology, Shenzhen) and characterized by Raman spectroscopy in our laboratory. For the target molecules, naphthalene, anthracene, phenanthrene, and pyrene were purchased from Sigma-Aldrich, Shanghai; perylene was purchased from Energy Chemical, Shanghai; tetracene and pentacene were purchased from TCI (Shanghai) Development Co. Ltd.; and benzoperylene and anthanthrene were purchased from J&K Scientific Ltd., Beijing. The solvent of decane was purchased from Tokyo Chemical Industry Development Co. Ltd., Shanghai. In all the XPBJ experiments, the target molecules were prepared to be 0.03 mM.

### Fabrication and conductance measurements

The single-molecule conductance measurements were performed using the XPBJ technique with a home-built setup at room temperature as described in previous reports (*29*). For further details, see section S1.

### Theoretical methods

Geometrical optimizations were performed using the standard Kohn-Sham self-consistent density functional code SIESTA, with vdW-DF functional of Dion *et al.* (*41*) with exchange modified by Berland and Hyldgaard (*42*), and a double ζ-polarized atomic-orbital basis set for carbon and hydrogen. The cutoff energy was 200 rydberg, and the force tolerance was 0.02 eV/Å. To compute their electrical conductance, the PAHs were each placed between two graphene electrodes. For each structure, the transmission coefficient *T*(*E*) describing the propagation of electrons of energy *E* from lead 1 to lead *j* (*j* = 2, 3, 4) was calculated using the Gollum quantum transport code, which uses the DFT mean-field Hamiltonian and overlap matrices from SIESTA and computes *T*(*E*) via the following formula

$$T(E)=\mathrm{Tr}[\Gamma_{1}(E)g(E)\Gamma_{j}(E)g^{†}(E)]$$ (1)

where $g(E)={\left(g_{0}^{-1}-\Sigma_{1}-\Sigma_{2}-\Sigma_{3}-\Sigma_{4}\right)}^{-1}$ is the retarded Green’s function in the presence of the electrodes and Γ*_j_*(*E*) = *i*(Σ*_j_*(*E*) − Σ*_j_*^†^(*E*))/2 is the anti-Hermitian part of the self-energies Σ*_j_*, which encode the electronic structure of the semi-infinite electrodes and molecule and the electrode-molecule interface. Γ*_j_* determines the broadening of transmission resonances due to the contact between the molecule and electrode *j*. *g*_0_ is the Green’s function of an isolated molecule. Last, the transmission coefficient between lead 1 and lead *j*, *T*_1*j*_(*E*), was obtained for electrons of energy *E* traveling separately from lead 1 to any of the other leads (*j* = 2, 3, 4).

The finite-temperature conductance is obtained from the transmission coefficient via the following formula

$$G=G_{0}\int_{-\infty }^{+\infty }\mathrm{dE}\;T(E)(-\frac{\partial f(E)}{\partial E})$$ (2)

where *G*_0_ = 2*e*^2^/*h* is the conductance quantum, *h* is the Planck’s constant, *e* is the charge of an electron, *f*(*E*) = (1 + exp ((*E* − *E*_F_)/*k*_B_*T*))^−1^ is the Fermi-Dirac probability distribution function, *E*_F_ is the Fermi energy, *T* is the temperature, and *k*_B_ is Boltzmann’s constant.

If the binding energy of one stacking interaction is $E_{i}^{b}$, then the probability of obtaining such a molecular location and orientation is $P_{i}=A^{-1}e^{-E_{i}^{b}/k_{B}T}$, where $A=\sum_{i}e^{-E_{i}^{b}/k_{B}T}$ is the partition function. As well known from statistical mechanics, if *G_i_* is the corresponding conductance, then the Boltzmann average of the conductance is *G* = ∑*_i_P_i_G_i_*. For each molecule, the transmission coefficient was computed for AA stacking, AB stacking, and one intermediate stacking (fig. S12). Last, the average conductance, weighted by the Boltzmann distribution based on binding energies of each junction configuration, is obtained from above equations or eqs. S2 to S6 (section S6).

## Supplementary Material

aba6714_SM.pdf

## SUPPLEMENTARY MATERIALS

Supplementary material for this article is available at http://advances.sciencemag.org/cgi/content/full/6/22/eaba6714/DC1
