# Supplementary material for: Cross-plane transport in a single-molecule two-dimensional van der Waals heterojunction
Source: Sci Adv. 2020 May 29;6(22):eaba6714. doi: 10.1126/sciadv.aba6714 (PMC7259930; doi:10.1126/sciadv.aba6714)
Supplement: aba6714_SM.pdf [file aba6714_SM.pdf]

Science Advances
AAAS

advances.sciencemag.org/cgi/content/full/6/22/eaba6714/DC1

Supplementary Materials for

Cross-plane transport in a single-molecule two-dimensional van der Waals heterojunction

Shiqiang Zhao, Qingqing Wu, Jiuchan Pi, Junyang Liu, Jueting Zheng, Songjun Hou, Junying Wei, Ruihao Li, Hatef Sadeghi, Yang Yang*, Jia Shi, Zhaobin Chen, Zongyuan Xiao, Colin Lambert*, Wenjing Hong*

*Corresponding author. Email: whong@xmu.edu.cn (W.H.); c.lambert@lancaster.ac.uk (C.L.); yangyang@xmu.edu.cn (Y.Y.)

Published 29 May 2020, *Sci. Adv.* **6**, eaba6714 (2020)
DOI: 10.1126/sciadv.aba6714

This PDF file includes:

Sections S1 to S6
Figs. S1 to S16
Tables S1 to S3

Section S1. Preparation of the graphene chip

To prepare the graphene chip, two graphene-coated copper wires were fixed on the surface of a stainless steel sheet with resin. The height of the resin is considerably larger than the diameters of the copper wire, thus the copper wires are freestanding. There is a height difference between the surface of the stainless steel and the copper wire, indicating that the copper wire is separated from the steel plate (see Fig. S1A). The diameter of the copper wire is 0.3 mm. Fig. S1 shows the images of a typical graphene chip. The separation between the two resin drops was controlled to be as small as 10-15 μm. The two electrodes should not contact to each other, otherwise the graphene layer may be destroyed before the break junction operation.

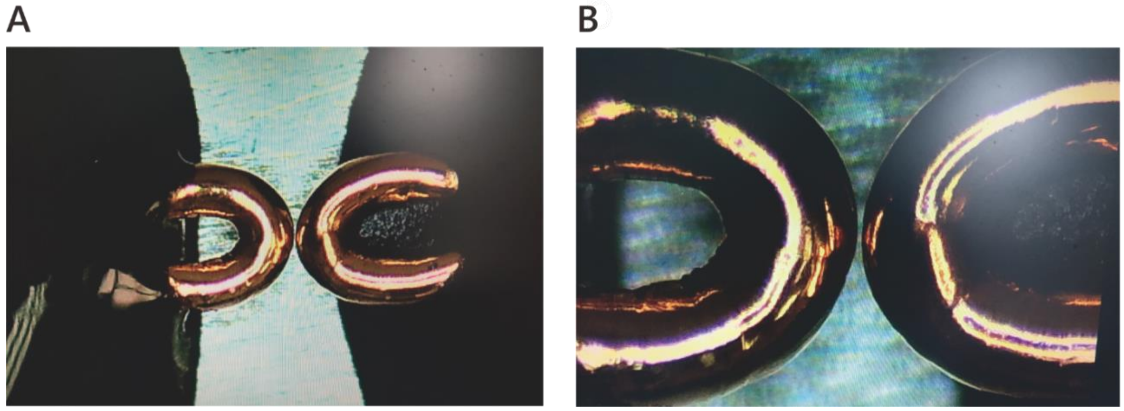

Fig. S1. Graphene chip. (**A**) Photos of a typical graphene chip. (**B**) Zoom-in of the central part of the chip, showing a micrometer scale separation between the two electrodes. (Photo credit: Jiuchan Pi, Xiamen University)

Section S2. Cross-plane break junction setup

In order to fabricate graphene-molecule-graphene two-dimensional van der Waals heterojunctions (graphene M-2D-vdWHs), we made significant modifications to the mechanically controllable break junction (MCBJ) technique to develop a novel cross-plane break junction (XPBJ) technique. A2 gives the photos of our XPBJ setup. In the conventional MCBJ technique, there is a notched wire fixed on the surface of an elastic substrate, while in our XPBJ technique it was instead by the aforementioned Cu wires pairs. Thus in order to form a nanogap that is comparable to the size of target molecules, the mechanic of the break junction technique is redesigned.

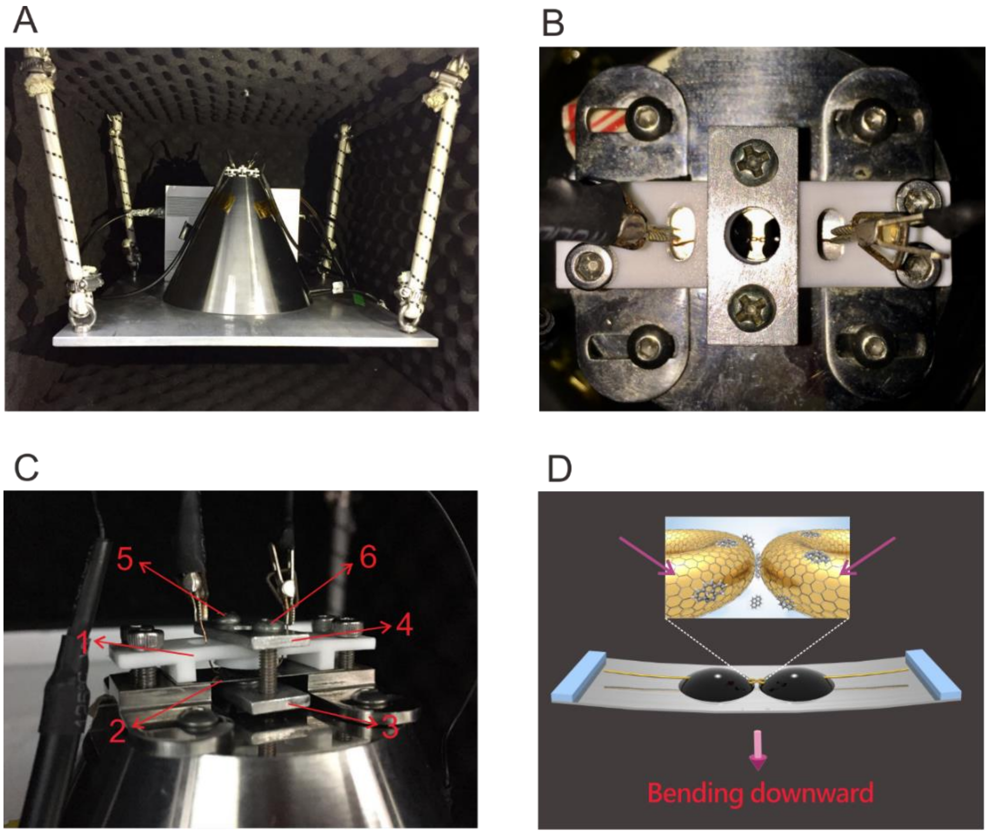

Fig. S2. Photos and a schematic diagram of the XPBJ setup. (**A**) A photo showing the overview of the XPBJ setup. (**B**) Top-view of the XPBJ setup. (**C**) Side-view of the XPBJ setup, where **1** is the liquid cell, **2** is the graphene chip, **3** and **4** are the two alloy plates, **5** and **6** are the two screws acting as adapting pieces. (D) a schematic drawing showing the downward bending of the graphene chip. (Photo credit: Jiuchan Pi, Xiamen University)

Fig. S2A shows the profile of our XPBJ setup, while Fig. S2, B and C gives the top-view and side-view of it, respectively. The pedestal of the XPBJ was processed to be a cone and placed onto a hanging platform, in order to reach high stability. A liquid cell made of PTFE and an O-ring made of FFKM (Perfluoroelastomer) (see Fig. S2C) were placed onto the surface of the graphene chip. Before the break junction experiment, the liquid cell and O-ring were soaked in piranha solution, boiled in distilled water, and dried by nitrogen flow. The solution was added to the liquid cell during the experiment, while the O-ring prevents the leakage of solution. Both of the liquid cell and the graphene chip were fastened with two aluminum alloy plates, while the bottom aluminum alloy plate was attached to the underneath actuator. In our break junction experiments, the actuator is composed of a stepping motor and a piezo stack. The stepping motor serves for the

coarse and quick adjustment, while the piezo stack serves for the more precise adjustment. By employing two screws as adapting pieces, the separation between the bottom aluminum alloy plate and the upper aluminum alloy plate was constant. When the underneath actuator moved downwards, the upper plate was brought down and pressed both the liquid cell and the graphene chip. The graphene chip was thus bent downward, resulting in a reduced nanogap that fits with the target molecule (see Fig. S2D).

During the break junction operation, i.e., the repeated opening and closing of nanogaps, the target molecules are stored in a liquid cell that is equipped on the surface of the chip (see Fig. S2C). In order to reduce the leakage current, in the present study, we utilised a solvent with weak polarity, i.e., decane, to dissolve the target molecules. Then the graphene-molecule-graphene junctions were repeatedly formed by continuously downward pulling and upward pushing of the substrate by a piezo actuator. The travel range of the piezo actuator is determined by an electrical feedback loop. Specifically, the moving direction of the piezo actuator was switched when the monitored conductance reaches the high conductance value of $10^{-2.5}$ G_0 or the low conductance value of $10^{-7.5}$ G_0. The preset high value prevents the possible damage of graphene layer or the formation of Cu-Cu contact, while the low value prevents the recording of insignificant noise data. During the break junction operation, the evolution of conductance was monitored by a home-built *I-V* converter with a sampling rate of 20 *k*Hz.

Section S3. Single-molecule conductance characterization

For each target molecule, ~1000 individual conductance traces were recorded, and all of them were adopted for the following data analysis without any selection. As shown in Fig. 1, F and G of the main text, for each graphene M-2D-vdWH, a pronounced peak emerged in the one-dimensional conductance histogram and we give each histogram separately (Fig. S3). To determine the most probable conductance value, the peaks were analysed by Gaussian fitting, and the generated parameters were listed in Table S1.

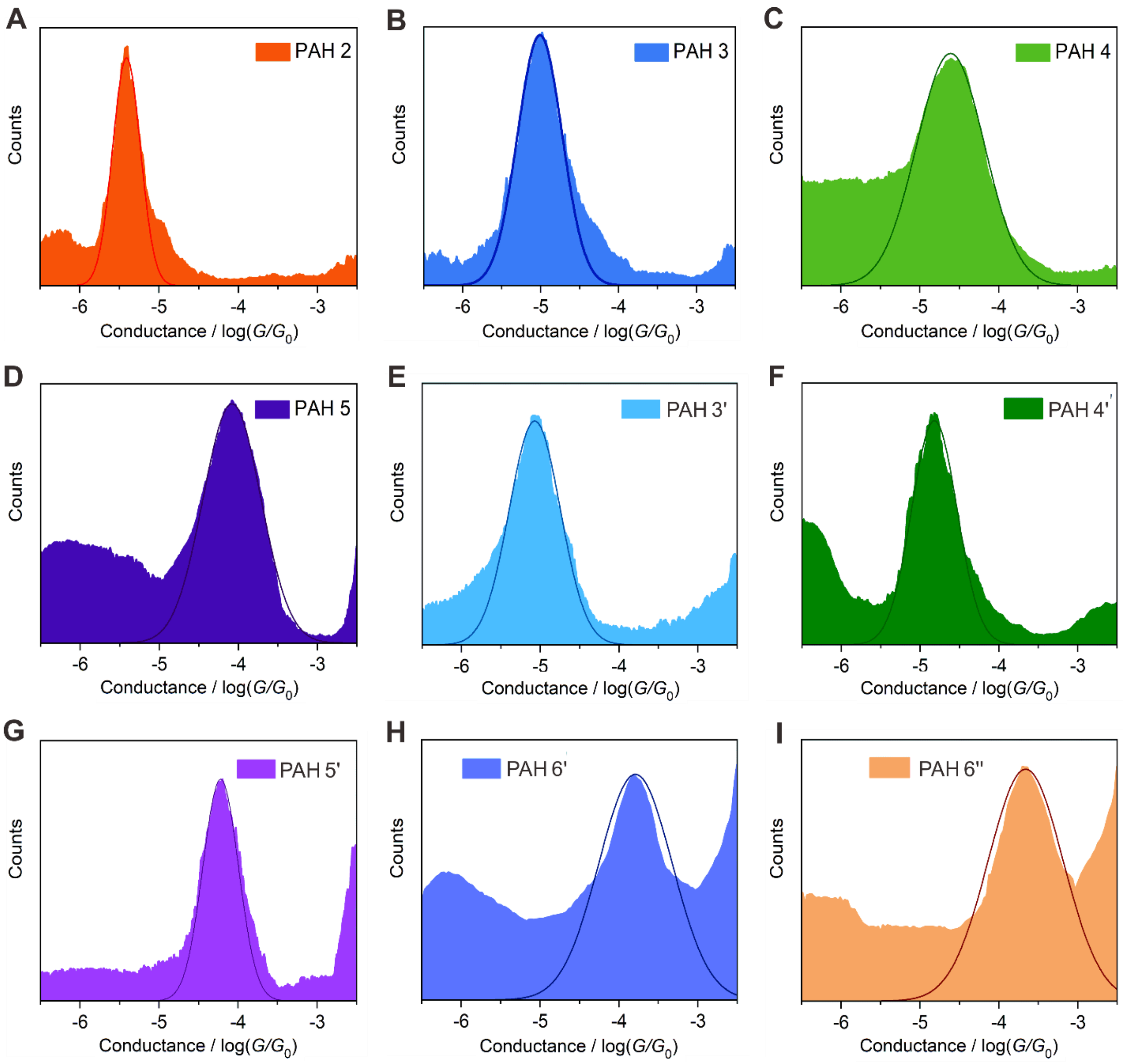

Fig. S3. The one-dimensional conductance-displacement histogram for the solutions containing PAHs

Table S1. Generated parameters of the one-dimensional histogram of the graphene M-2D-vdWHs.

Molecule	Center / log(G/G_0)	Sigma	FWHM
Naphthalene (PAH 2)	-5.41	0.175	0.412
Anthracene (PAH 3)	-5.01	0.285	0.671
Tetracene (PAH 4)	-4.61	0.450	1.060
Pentacene (PAH 5)	-4.08	0.365	0.860
Phenanthrene (PAH 3')	-5.07	0.275	0.648
Pyrene (PAH 4')	-4.82	0.275	0.648
Perylene (PAH 5')	-4.18	0.105	0.247
Benzoperylene(PAH 6')	-3.79	0.525	1.236
Anthanthrene (PAH 6'')	-3.66	0.490	1.154

The two-dimensional conductance-displacement histograms were generated by overlapping all individual traces. The individual traces are aligned with a relative zero, that is, the piezo distance is regarded as zero as the conductance value is $10^{-3.0}$ G_0. Then the data density in each bin was counted to get the two-dimensional conductance-displacement histograms. For both of the one-dimensional conductance histograms and the two-dimensional conductance-displacement histograms, conductance values ranged from $10^{-2.5}$ G_0 to $10^{-7.5}$ G_0 as determined by the travel range of piezo actuator. Fig. S4 gives the two-dimensional conductance-displacement histogram for the pure solvent of decane.

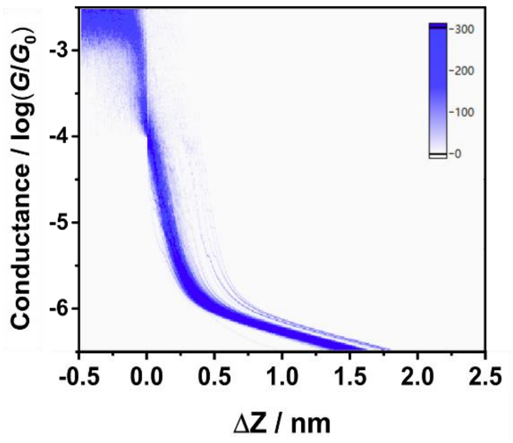

Fig. 4. The two-dimensional conductance-displacement histogram for the pure solvent of decane.

Section S4. Molecular length characterization with STM-BJ and MCBJ

In order to determine the molecular length, we adopted a “graphene STM-BJ” protocol to calibrate the stretching distance in pure decane without target molecules (see Fig. S5). We used the same graphene-coated copper wire as the STM tip to vertically approach the copper substrate with graphene on it. Since the piezo stack stretched linearly was driven by the applied piezo voltage, the relative displacement of this vertical graphene electrode driven by piezo stack will be calculated through the recorded piezo voltage changes. As the data acquisition setup recorded the conductance value at each corresponding piezo voltage, therefore, the relative piezo voltage changes can be calculated and summarized when driving the electrode from the conductance value of $10^{-3.2}$ G_0 to $10^{-5.2}$ G_0 repeatedly. Fig. S5A showed the obtained histogram of piezo voltage changes of over 1000 individual cycles with fitted peak centre of 9.89×10^{-4} V. Thus, as the piezo voltage enables a 9.7 μm distance range within 150 V, the relative displacement of the graphene electrode in pure decane solvent during the linear conductance range of $10^{-3.2}$ G_0 to $10^{-5.2}$ G_0 will be 0.27 nm (Fig. S5B). Fig. S5B gives the two-dimensional conductance-displacement histogram and piezo distance statistics for decane solution. Therefore, to calibrating the electrode stretching rate in MCBJ for decane solvent, we used the same statistical length of 0.27 nm for the conductance of the decane solvent from $10^{-3.2}$ G_0 to $10^{-5.2}$ G_0, since the tunneling decay constant of β is almost the same between XPBJ and graphene STM-BJ scenarios. Therefore, when adding molecular solution instead of the pure solvent, the relative displacement distribution calibrated in same

stretching rate will be in accord with the length of molecular junction. For example, in the system for solutions containing PAH 3, when the decane solvent is 0.27 nm in the conductance range of $10^{-3.2}$ G_0 to $10^{-5.2}$ G_0, the plateau length of PAH 3 in its conductance range of $10^{-4.2}$ G_0 to $10^{-5.5}$ G_0 at the same stretching rate is 1.02 nm (Fig. 2, A and B). Fig. S11 shows the result of plateau length analysis for the systems of solutions containing PAHs respectively. The conductance range selected for the plateau length analysis ranges for PAH 2 is between $10^{-4.7}$ G_0 to $10^{-5.8}$ G_0, PAH 4 is between $10^{-3.9}$ G_0 to $10^{-5.5}$ G_0, PAH 5 is between $10^{-3.4}$ G_0 to $10^{-4.9}$ G_0, PAH 3' is between $10^{-4.5}$ G_0 to $10^{-5.5}$ G_0, PAH 4' is between$10^{-4.1}$ G_0 to $10^{-5.4}$ G_0, PAH 5' is between $10^{-3.5}$ G_0 to $10^{-4.5}$ G_0, PAH 6' is between $10^{-3.0}$ G_0 to $10^{-4.5}$ G_0, and PAH 6'' is between $10^{-3.0}$ G_0 to $10^{-4.5}$ G_0.

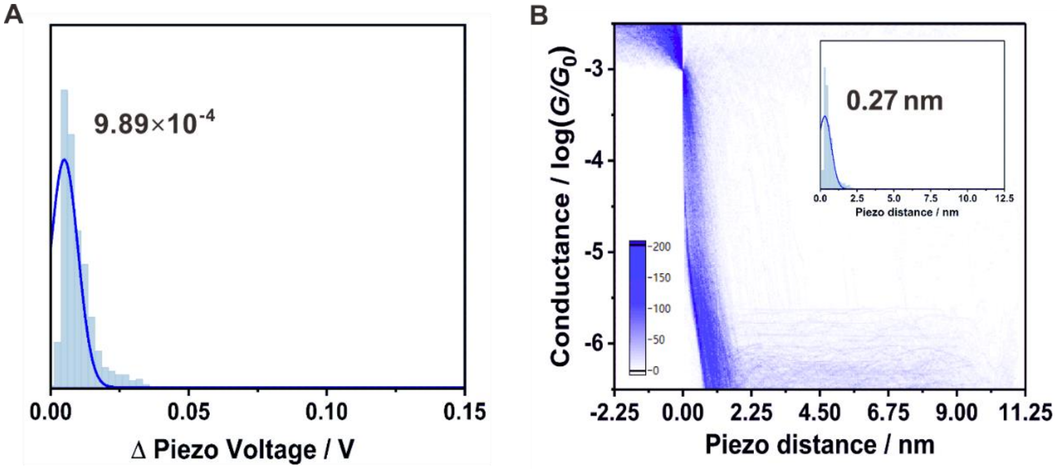

Fig. S5. The two-dimensional conductance-displacement histograms and plateau length analysis for decane solution by STM-BJ.

To determine the most probable plateau length value, the two-dimensional conductance-distance histogram and plateau length of the PAHs were measured (see Fig. S7). In the distribution histogram of plateau length, the peaks were analysed by Gaussian fitting, and the generated parameters were listed in Table S2. It was found the plateau length of PAHs lies in a range from 0.87 to 1.08 nm. According to the previous report (*Nature* 567, 323-333, 2019), the vdW distance and calculated vdW gap between the two graphene layers were 0.34 nm and 0.20 nm. For the in-plane charge transport mode, the stacking pattern between single PAH molecule and graphene electrode pairs is the face to face one (see Fig. S6D), while the stacking pattern for cross-plane charge transport is point to face one (see Fig. S6E). For these two configurations, the spacings of the two graphene layers incorporated with PAH 5 were calculated to be 0.68 nm (face to face) and

2.09 nm (point to face). It was found the spacing of point to face was much larger than that of the experiment result (0.99 nm). Moreover, all the plateau length of the PAHs measured are approximate 1.0 nm. These findings further suggested that in our experiments the stacking pattern between single PAH molecule and graphene electrode pairs was face to face.

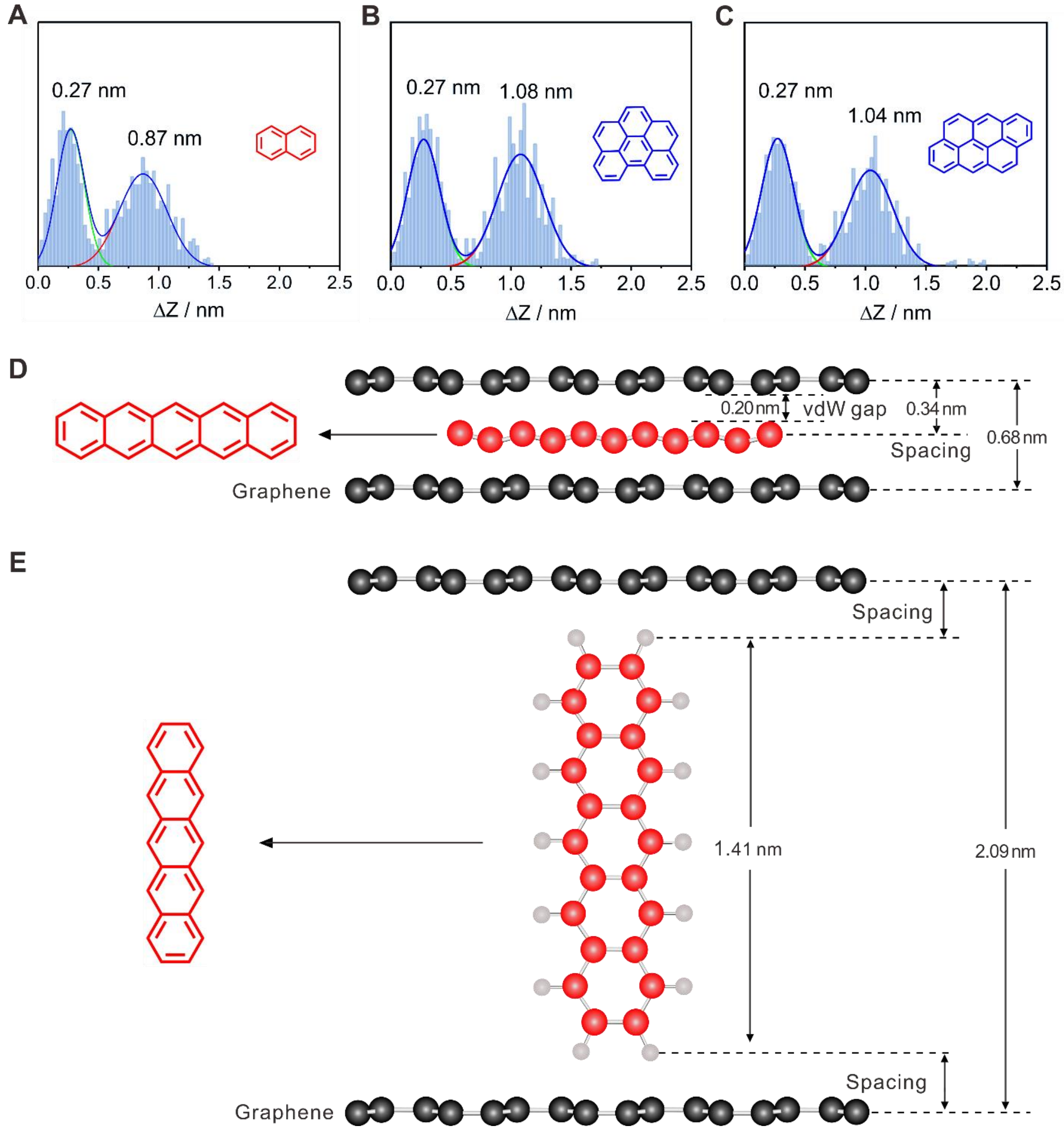

Fig. S6. Configuration analysis for the as-fabricated graphene M-2D-vdWHs. (**A**) Distribution of plateau length for PAH 2. (**B**) Distribution of plateau length for PAH 6'. (**C**) Distribution of plateau length for PAH 6''. Analysis on the spacings of the two graphene layers incorporated with PAH 5 as the stacking pattern is either face to face (**D**) or point to face (**E**).

Table S2. Generated parameters of plateau length analysis of the graphene M-2D-vdWHs.

Molecule	Center / nm	Sigma	FWHM
**Naphthalene (PAH 2)**	**0.87**	0.198	0.467
**Anthracene (PAH 3)**	**1.02**	0.119	0.281
**Tetracene (PAH 4)**	**1.05**	0.195	0.460
**Pentacene (PAH 5)**	**0.99**	0.214	0.505
**Phenanthrene (PAH 3')**	**0.93**	0.218	0.514
**Pyrene (PAH 4')**	**0.91**	0.260	0.613
**Perylene (PAH 5')**	**1.03**	0.151	0.354
**Benzoperylene (PAH 6')**	**1.08**	0.195	0.458
**Anthanthrene (PAH 6'')**	**1.04**	0.188	0.443

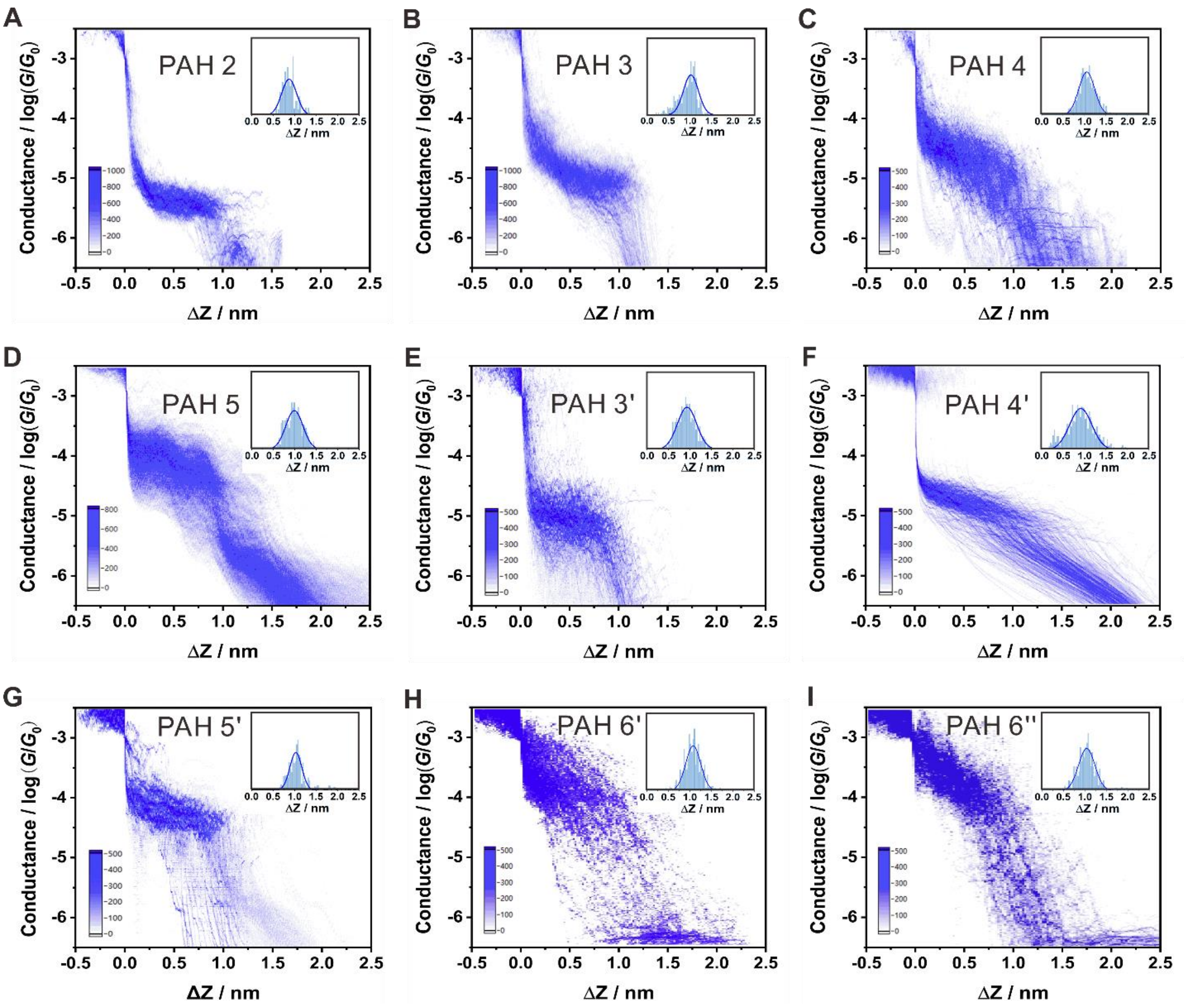

Fig. S7. The two-dimensional conductance-displacement histograms for the solutions containing PAHs and histograms of the molecular length with a Gaussian fit.

To further demonstrate the reproducibility of the geometry, we provided more data below (Figs. S8 and S9) from several independent experiments of nine probe molecules. It can be found that they show the result are consistent with the results above, which confirms the reproducibility of our method.

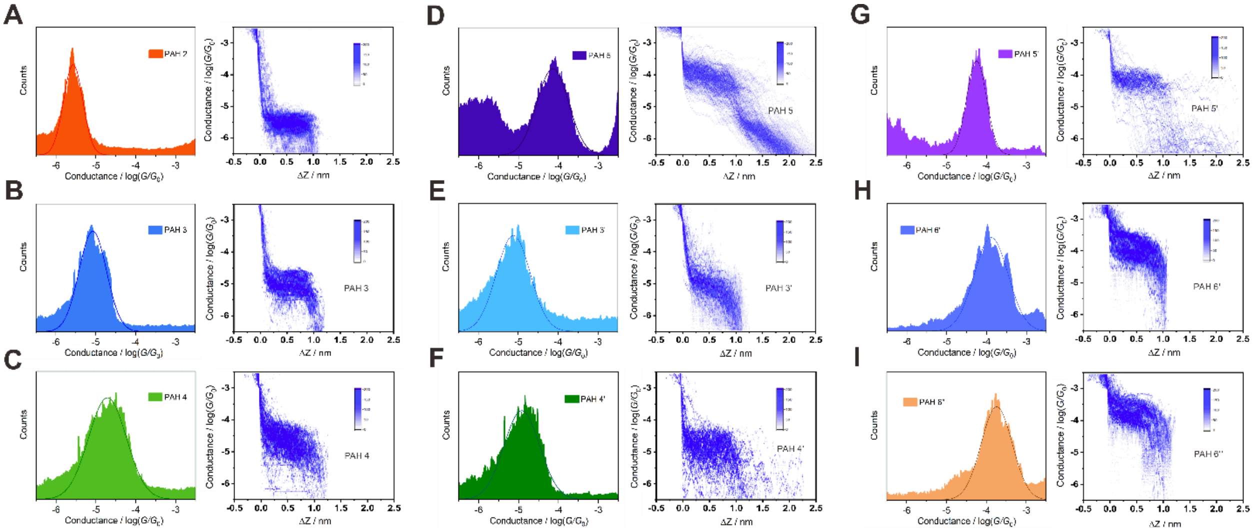

Fig. S8 The one-dimensional conductance histograms and two-dimensional conductance-displacement histograms for graphene M-2D-vdWHs fabricated by the second batch of microchips. Nine probe molecules (**A-I**) are PAH 2, PAH 3, PAH 4, PAH 5, PAH 3', PAH 4', PAH 5', PAH 6', and PAH 6", respectively. All the data were collected from independent microchips and experiments.

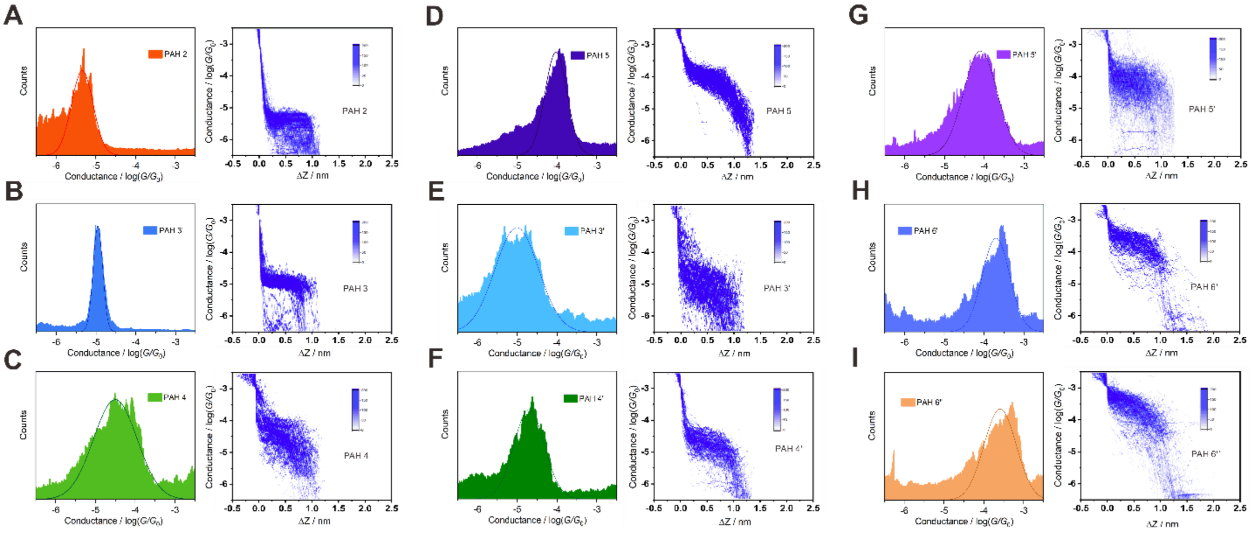

Fig. S9 The one-dimensional conductance histograms and two-dimensional conductance-displacement histograms for graphene M-2D-vdWHs fabricated by another batch of microchips. Nine probe molecules (**A-I**) are PAH 2, PAH 3, PAH 4, PAH 5, PAH 3', PAH 4', PAH 5', PAH 6', and PAH 6", respectively. All the data were collected from independent microchips and experiments.

To study the influence of concentration on the single-molecule conductance measurement, two-dimensional conductance-displacement and one-dimensional conductance histograms for the solutions containing PAH 4' with 0.01 mM, 0.02 mM, 0.03 mM, 0.04 mM, and 0.05 mM were conducted, respectively. In the two-dimensional conductance-displacement (see Fig. S10A), the curves in the green rectangular dotted frame show direct tunnelling (T) behaviour, while the curves in red elliptical dotted frame show molecule-mediated transport (M) behaviour. With the concentration increases, the intensity of the T component decreases, while the intensity of the M component increases. This observation shows that the formation probability of graphene M-2D-vdWH is dependent on the concentration. In contrast, in the one-dimensional conductance histograms, there is no discernable variation for the position of the conductance peak. Thus, in our experiments the measured conductance value is independent on the concentration, demonstrating that the conductance characterization comes from single probe molecule.

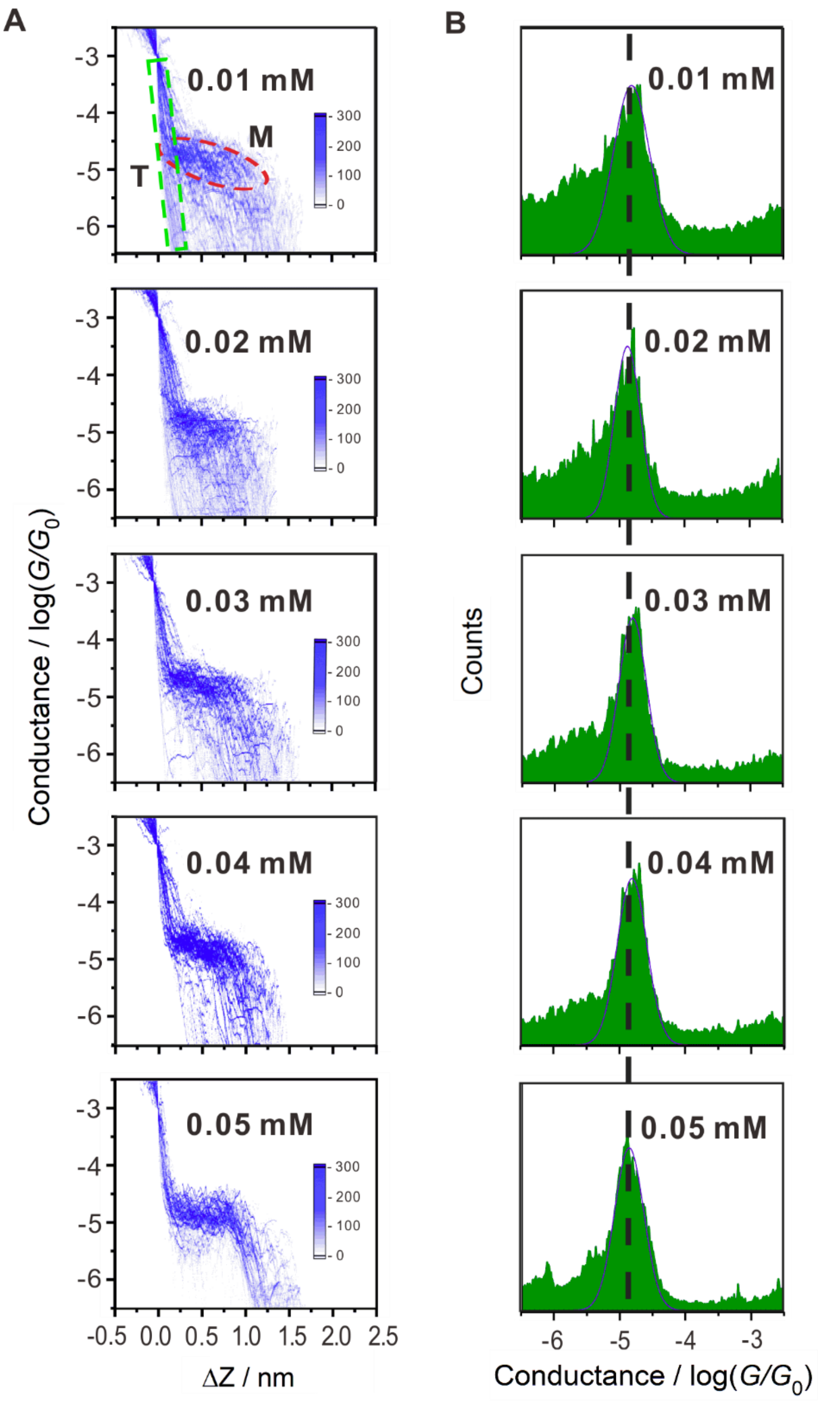

Fig. S10. The one-dimensional conductance and two-dimensional conductance-displacement histograms for the solutions containing PAH 4' with different concentrations.

Section S5. Supplementary for Raman characterization

In our study, Raman characterization was carried out by iDSpec ARCTIC (NCS Testing

Technology, Beijing). All the measurements were carried out under a confocal spectrometer (×50 objective) with a 600 lines / mm grating and a 632.8 nm excitation. The Raman spectra of graphene electrodes were acquired with a 4.96 mW laser power and an integration time of 10 s. For PAHs in the powder states, the Raman spectra were acquired with a 0.39 mW laser power and an integration time of 5 s.

Fig. S11A shows the Raman spectrum of the prepared graphene electrode before XPBJ operation. We analysed the G and 2D band of graphene before and after XPBJ by Gaussian fitting, and the FWHM were listed in Table S3. Fig. S11, B and C shows the Raman spectra of the PAHs in the powder states, as well as the corresponding Raman spectra of the established graphene-PAH-graphene 2D-vdWHs. As highlighted by the pink line, the peak of 1529 cm^{-1} that observed in the spectra of graphene-PAH-graphene 2D-vdWHs, is absent in the spectra that collected from the PAHs in the powder states. Considering the fact that neither the graphene nor the alone target molecule exhibits such a peak, it is inferred that the peak of 1529 cm^{-1} comes from the adsorption of the PAHs on the graphene layer.

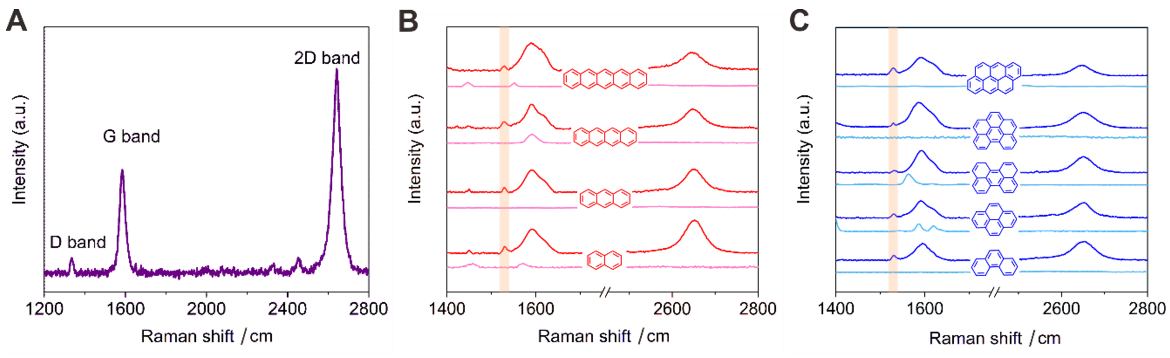

Fig. S11. Raman characterization of the graphene chip, graphene-molecule-graphene 2D-vdWH devices, and the corresponding PAHs in the powder states. (**A**) Raman spectrum of the prepared graphene electrode. (**B, C**) Raman spectra of the graphene-molecule-graphene 2D-vdWH devices with different PAHs. In each panel, the upper spectrum (Red and blue lines) was collected from the graphene M-2D-vdWH, while the bottom one (light red and light blue) is from the corresponding PAHs in the powder states.

Table S3. FWHM of the G and 2D band.

Sample	G band FWHM	2D band FWHM
Before XPBJ	33.804	48.497
After XPBJ	31.072	56.493

Section S6. Supplementary for theoretical calculation

The transmission spectra *T(E)*, probability P_i, electrical conductance *G*, and the Boltzmann-weighted average conductance $G(E_F)$ based on the above binding energies E_i^b between the single PAH molecule and graphene sheet can be written as follows:

$$\mathrm{T(E) = Tr\left[\Gamma_L(E)G(E)\Gamma_R(E)G^{\dagger}(E)\right]} \quad (1)$$

$$P_i = \frac{1}{A}e^{-E_i^b/(k_BT)} \quad (2)$$

$$A = \sum_{i=1}^{N} e^{-E_i^b/(k_BT)} \quad (3)$$

$$\mathrm{G = G_0L_0} \quad (4)$$

$$G(E_F) = \sum_{i=1}^{N} G_i(E_F)P_i \quad (5)$$

$$\mathrm{L_n} = \int_{-\infty}^{+\infty} \mathrm{dE(E - E_F)^n T(E)\left(-\frac{\partial f(E)}{\partial E}\right)} \quad (6)$$

Where T is the temperature and k_B is Boltzmann's constant, L_0 is the value of L_n when n=0.

Fig. S12 shows binding energy results for the target PAH molecules and graphene electrodes. For each molecule, three configurations are taken into consideration, denoted as 'AA', 'mid', and 'AB' in Fig. S12, A-D, where PAH 3 is shown as an example. Specifically, 'AA' and 'AB' represent AA and AB stacking between the target molecule and the two graphene sheets, while 'mid' indicates a configuration between 'AA' and 'AB'. In order to distinguish the molecule from the graphene, blue and grey colours are used for the carbon atoms, while the white colour is for hydrogen.

To obtain the binding energy E_b, basis set superposition error (BSSE) correction is utilised, where E_b=E[AB]- E[Ab]- E[aB]. Fig. S13 shows binding energies of the three configurations for

each PAH molecule on a graphene sheet, as a function of the perpendicular distance between the two parallel planes containing the molecule and the graphene sheet. The red, blue, magenta dot lines represent the results for ‘AA’, ‘mid’, ‘AB’, respectively. From the curves, it is obvious that ‘AB’ configurations, as shown by the red curves, are the most favourable structures, because they possess the lowest binding energies. The ‘AB’ structures are most stable when the distance between molecule and graphene is around 3.3~3.4 Å, while for ‘AA’ stacking, the optimum distance is around 3.5 Å.

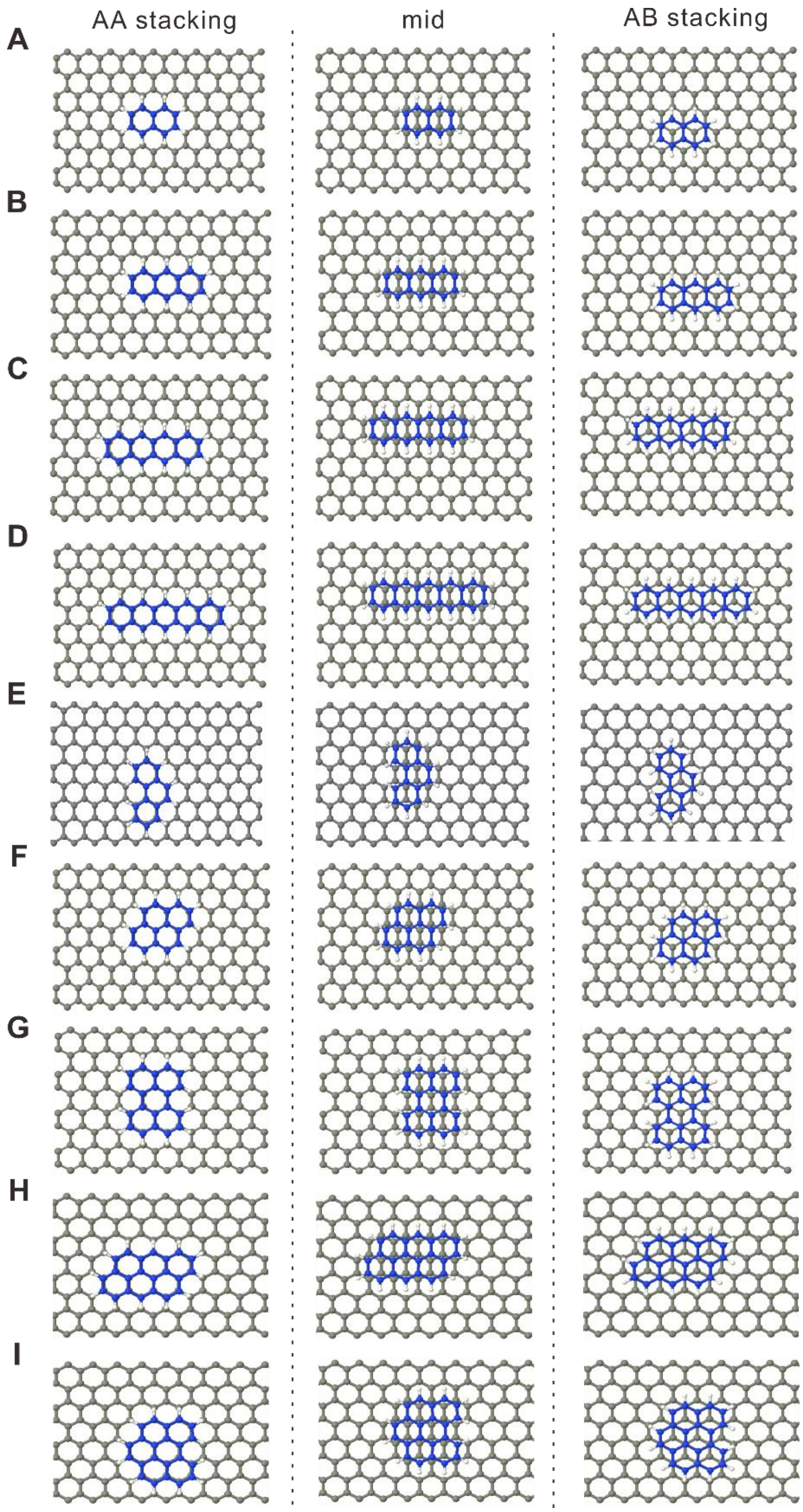

Fig. S12. Configurations between graphene and the PAH molecules.

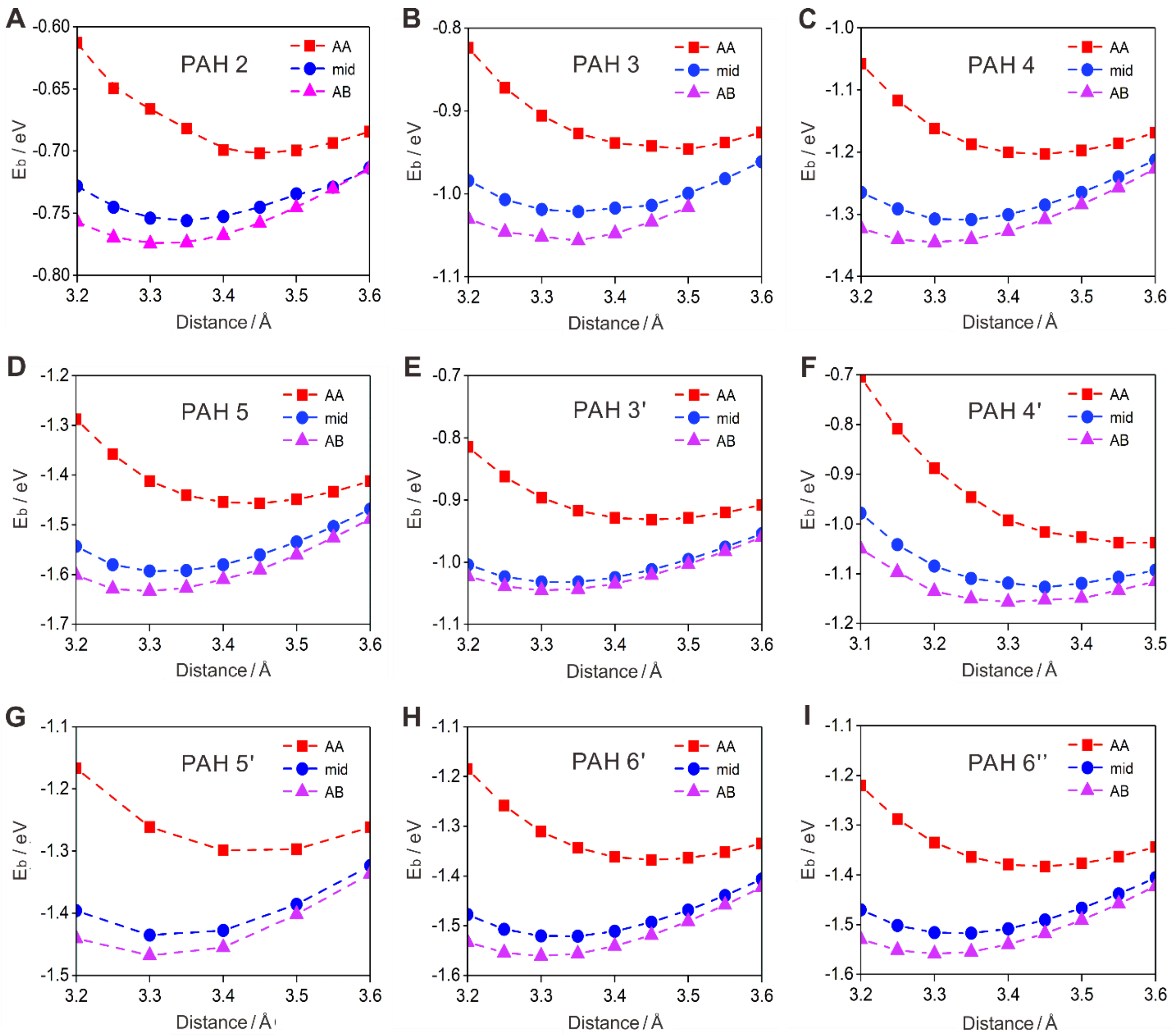

Fig. S13. The corresponding binding energies E_b versus the distance between graphene and the target PAH molecules.

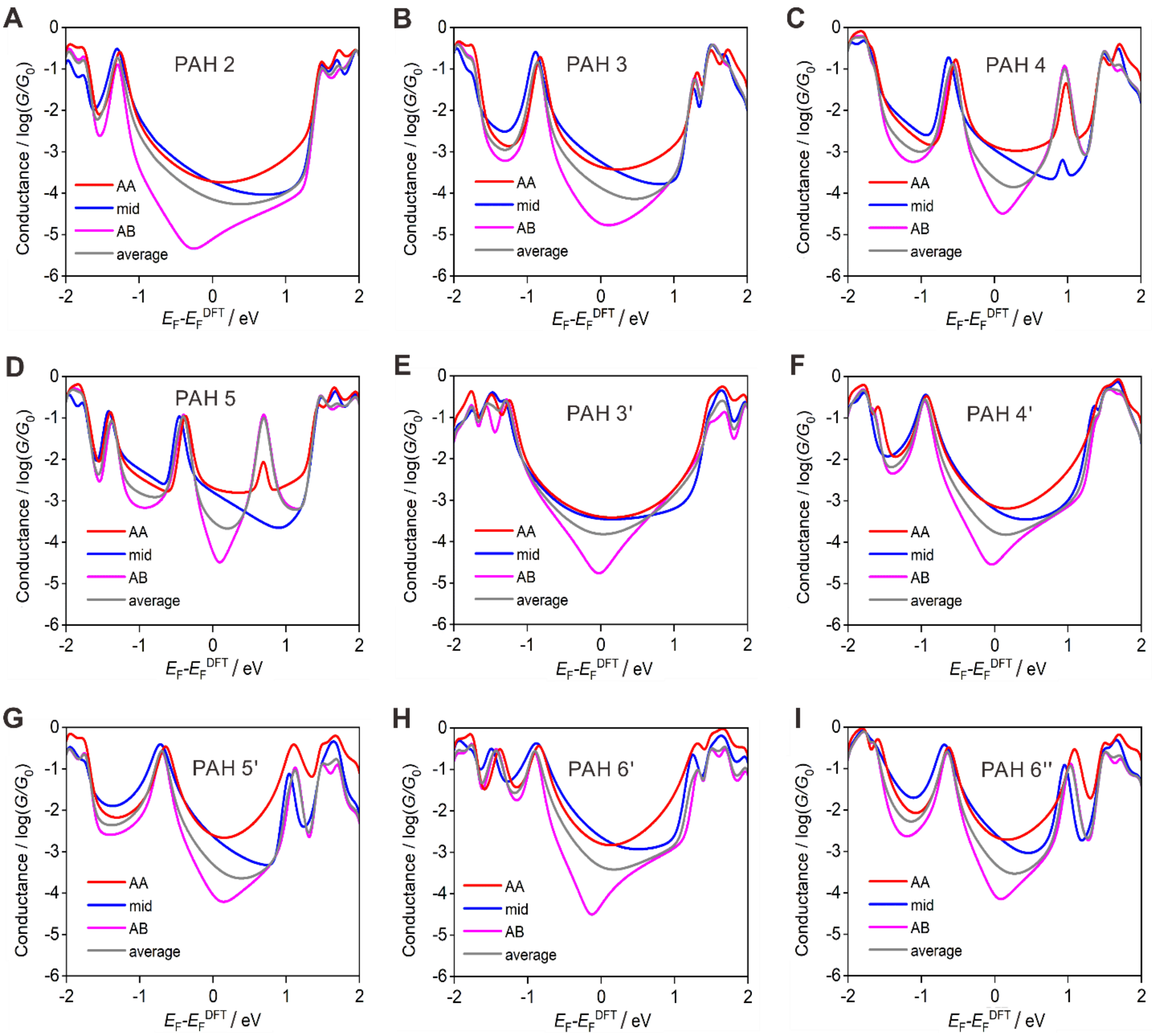

Fig. S14. Conductance variations as the function of the Fermi level. (**A-I**) Conductance originating from the electron transport functions from lead 1 to lead 3 and lead 4 for the target molecules with three binding configurations at room temperature. The grey curves present the Boltzmann-weighted average conductance with Boltzmann factors obtained using the above binding energies for the PAH molecules.

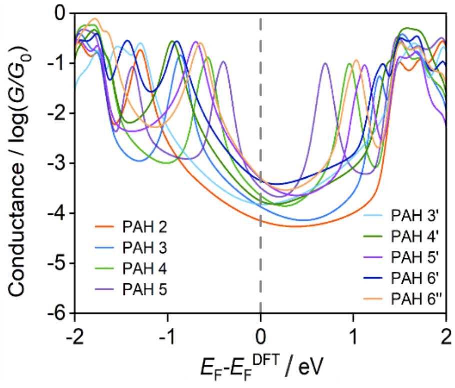

Fig. S15. Conductance spectra as the function of the Fermi level for the PAH molecules.

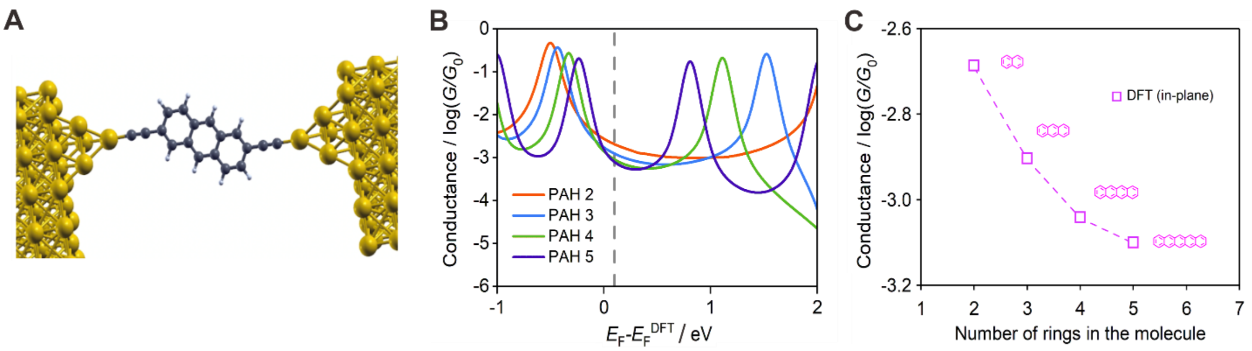

Fig. S16. Transport properties of PAH molecules in traditional gold-gold junctions with in-plane transport. (**A**) Schematic of in-plane transport where one PAH molecule (e.g. PAH 3) are sandwiched in gold-gold junction by carbon-carbon triple bond on each side. (**B**) Conductance spectra as the function of the Fermi level relative to that estimated by DFT. The gray dashed vertical line shows one possible Fermi energy (0.1eV). (**C**) Conductance evolution at the chosen Fermi energy in **B**.
